# Viral Infection of Human Lung Macrophages Increases PDL1 Expression via IFNβ

**DOI:** 10.1371/journal.pone.0121527

**Published:** 2015-03-16

**Authors:** Karl J. Staples, Ben Nicholas, Richard T. McKendry, C. Mirella Spalluto, Joshua C. Wallington, Craig W. Bragg, Emily C. Robinson, Kirstin Martin, Ratko Djukanović, Tom M. A. Wilkinson

**Affiliations:** 1 Academic Unit of Clinical and Experimental Sciences, University of Southampton Faculty of Medicine, Sir Henry Wellcome Laboratories, Southampton General Hospital, Southampton, United Kingdom; 2 Southampton NIHR Respiratory Biomedical Research Unit, Southampton General Hospital, Southampton, United Kingdom; Albany Medical College, UNITED STATES

## Abstract

Lung macrophages are an important defence against respiratory viral infection and recent work has demonstrated that influenza-induced macrophage PDL1 expression in the murine lung leads to rapid modulation of CD8+ T cell responses via the PD1 receptor. This PD1/PDL1 pathway may downregulate acute inflammatory responses to prevent tissue damage. The aim of this study was to investigate the mechanisms of PDL1 regulation by human macrophages in response to viral infection. *Ex-vivo* viral infection models using influenza and RSV were established in human lung explants, isolated lung macrophages and monocyte-derived macrophages (MDM) and analysed by flow cytometry and RT-PCR. Incubation of lung explants, lung macrophages and MDM with X31 resulted in mean cellular infection rates of 18%, 18% and 29% respectively. Viral infection significantly increased cell surface expression of PDL1 on explant macrophages, lung macrophages and MDM but not explant epithelial cells. Infected MDM induced IFNγ release from autologous CD8+ T cells, an effect enhanced by PDL1 blockade. We observed increases in PDL1 mRNA and IFNβ mRNA and protein release by MDM in response to influenza infection. Knockdown of IFNβ by siRNA, resulted in a 37.5% reduction in IFNβ gene expression in response to infection, and a significant decrease in PDL1 mRNA. Furthermore, when MDM were incubated with IFNβ, this cytokine caused increased expression of PDL1 mRNA. These data indicate that human macrophage PDL1 expression modulates CD8+ cell IFNγ release in response to virus and that this expression is regulated by autologous IFNβ production.

## Introduction

The optimal immune response to respiratory viral infections requires a coordinated and balanced response from an array of innate and adaptive responses. Although the influenza virus primarily infects the respiratory epithelium, this virus can also infect and replicate in human alveolar macrophages[1–3]. Macrophages are not only a key line of defence in the respiratory tract, responsible for phagocytosis and clearance of infectious organisms but are also orchestrators of the adaptive immune response through presentation of antigen and by the cytokines they release[4,5]. Direct infection of airway macrophages has been postulated as a key mechanism in the development of secondary bacterial infection of the airway leading to consequent pneumonia as described in 1918 influenza pandemic cases[3]. It has also been hypothesised that the mortality associated with novel strains of influenza (e.g. H5N1), is due to increases in macrophage cytokine release[6] highlighting a key role in the control of the inflammatory response.

Recent studies have suggested that macrophage expression of Programmed Death Ligand (PDL)-1 is important in regulating T cell responses to influenza infection[7]. PDL1 is the ligand for the Programmed Cell Death (PD) receptor 1, which is a member of the CD28 family of T cell receptors with CD80 and CD86 being ligands for CD28. In the standard model of T cell receptor (TCR) activation, activation of CD28 provides a necessary co-stimulation to prevent T cell anergy[8]. In contrast, binding of PDL1 to PD1 causes inhibition of TCR-mediated phosphatidylinositol-3-kinase (PI3Kinase) activation leading to inhibition of T cell proliferation and cytokine release[9]. The increased expression and activation of the PD1/PDL1 axis in chronic viral infections such as HIV and Hepatitis C (HCV) can lead to progressive loss of T cell function[10,11]. However, it was only recently that a role for this PD1/PDL1 pathway has been elucidated in the control of immune function in acute infections and increased PDL1 expression in response to pathogens was demonstrated to be crucial for impairment of CD8 cytotoxicity[7] and the development of regulatory T cells[12]. We have recently demonstrated that CD4 cytotoxic T cells play an important role in protecting against severe influenza infection. This work demonstrated an additional role for MHC class II expressing cells and T helper cell responses[13] highlighting the potential importance of macrophage-T cell interactions in the control of influenza infection.

Whilst PDL1 expression has been shown to be increased on human airway macrophages in response to respiratory viral infections[7], the mechanisms of the expression of this ligand on macrophages in response to influenza has not been fully elucidated. Therefore we used a validated *ex vivo* human lung model of influenza infection[14], where we have demonstrated infection of resected lung tissue using immunohistochemistry and flow cytometry, by the expression of viral nucleoprotein (NP)-1[14]. In this *ex vivo* model we have observed NP1 positivity only in epithelial cells and macrophages suggesting that these are the two major cell types for which the virus has tropism. In addition to explant tissue we have used lung macrophages and monocyte-derived macrophages (MDM)[15,16] to further explore the effects of influenza infection on macrophage expression of PDL1.

## Materials and Methods

### Ethics

The collection of lung tissue and blood was approved by and performed in accordance with the ethical standards of the Southampton and South West Hampshire Research Ethics Committee (LREC no: 09/H0504/109 for tissue and 08/H0504/138 for blood). Written informed consent was obtained from all participants.

### *Ex vivo* infection of lung parenchymal tissue

Resected human lung tissue was obtained from patients undergoing airway re-section surgery at our regional thoracic surgical unit. *Ex vivo* infection of resected lung tissue was carried out as previously described[14].

### Lung macrophage isolation

Parenchymal tissue, distant from the resection margin and any gross pathology, was dissected from the lobe and stored briefly in PBS (maximum 30 min) during transport to the laboratory and macrophages were isolated according to previously published protocols[38].

### Monocyte Isolation & differentiation

Monocytes were isolated from human peripheral blood mononuclear cells (PBMC) and differentiated into macrophages as previously described[17].

### Isolation of CD8+ T cells

CD8+ T cells were isolated from the monocyte-depleted PBMC suspension using CD8+ microbeads (Miltenyi-Biotec, Bisley, UK) according to the manufacturer’s instructions. CD8+ T cells that resulted from this separation step were frozen at -80°C in 10% (v/v) DMSO/HI-FBS for use in later ELISpot analysis.

### Infection of lung macrophages and MDMs

Influenza A virus strain X31 was supplied at a concentration of 4 x 10^7^ pfu/ml (a kind gift of 3VBiosciences). Inactivated virus (UVX31) was prepared by exposure of the X31 to an ultra-violet (UV) light source for 2 h. Macrophages were incubated for 2 h with no virus, or 4000 pfu (lung) or 500 pfu (MDM) of X31 or UVX31. Cells were then washed and incubated for a further 22 h at 37°C, 5% CO_2_. Supernatants were harvested for cytokine analysis, HA shedding and LDH assays. Cells were collected using non-enzymatic cell dissociation solution (Sigma, Poole, UK) for immediate flow cytometry analysis or lysed and stored at -80°C for RNA analysis.

A similar method was used to infect MDM with Respiratory Syncytial Virus (RSV—strain M37—Meridian Life Science Inc, Memphis, USA). Inactivated RSV (UV-RSV) was prepared by exposure of the X31 to an ultra-violet (UV) light source for 2 h. 500 μl of stock RSV (3.5 x 10^6^ pfu) was diluted 1:1 in basal RPMI; and a 1:10 dilution was made in each well and incubated for 2 h at 37°C. MDMs were then washed with basal RPMI and cultured in RS RPMI for a further 22 h.

### Flow cytometry analysis

Samples were resuspended in FACS buffer (PBS, 0.5% w/v BSA, 2 mM EDTA) containing 2 mg/ml human IgG before being incubated on ice in the dark for 30 min in the presence of fluorescently-labelled antibodies or isotype controls (all BD Biosciences, Oxford, UK). Intracellular staining for viral nucleoprotein (NP)-1, was performed using BD Cytofix/Cytoperm kit according to manufacturer’s instructions, and AlexaFluor 488 (AF488)-conjugated anti-NP1 antibody (HB-65, a kind gift of 3VBiosciences). Flow cytometric analysis was performed on a FACSAria using FACSDiva software v5.0.3 (all BD).

### ELISpot

MDM were either not infected, or were treated with 2.5 x 10^4^ pfu/ml X31 Influenza A H3N2 virus at 37°C for 2 h prior to loading on a 1-DIK antibody (MabTech, Stockholm, Sweden) coated 0.45 μm MultiScreen-IP Filter Plates (Millipore, Watford, UK) at a concentration of 5 x 10^4^ MDM/well. 2.5 x 10^5^ monocyte-depleted PBMC or 1 x 10^5^ CD8+ T cells were then added to MDM-containing wells in the presence or absence of anti-PDL1 or isotype control antibodies (10 μg/ml—eBiosciences) and incubated at 37°C for a further 22 h. ELISpot was then carried out according to manufacturer’s instructions (MabTech). In initial experiments, no IFNγ staining was seen in wells containing infected MDM or lymphocytes alone (data not shown). Peripheral blood T cells do not appear to be infected when exposed to X31 (S3 Fig.).

### siRNA experiments

After isolation, monocytes were cultured in GM-CSF media as described above for 11 d before cells were washed with basal RPMI. One hundred microliters of GM-CSF media was added to the cultures before incubating with 50 nM scrambled siRNA or siRNA specific to IFNβ using 6 μl HiPerFect (Qiagen, Manchester, UK) for 24 h in GM-CSF media. MDM were subsequently infected with influenza as previously described and IFNβ and PDL1 mRNA expression were analysed using RT-PCR.

### RNA Isolation & RT-PCR

RNA was extracted from macrophages using a Stratagene Microprep Kit. Reverse transcription was carried out using a nanoScript Reverse Transcriptase kit and PCR amplifications for cytokine gene expression (*IFNα*, *IFNβ*, *IFNγ*, *IL10*, *PDL1)* were performed. Gene expression was normalized to β_2_-microglobulin gene expression and quantified using the ΔΔ C_T_ method.

### Supernatant analyses

IFNβ concentrations in culture supernatants were measured by ELISA according to the manufacturer’s instructions (MSD, Gaithersberg, USA). Culture supernatants were analysed by Luminex assay as per manufacturer’s instructions (Bio-Rad). LDH release was measured using CytoTox 96 Non-Radioactivity Cytotoxicity Assay according to the manufacturer’s instructions (Promega, Southampton, UK). Release of viral hemagglutinin was measured using a dot blot assay on nitrocellulose membrane detected using a rabbit polyclonal anti-influenza serum. The bound rabbit antibodies were detected using the Bio-Rad anti-Rabbit HRP detection system according to the manufacturer’s instructions.

### Statistics

Statistical analyses were performed using a paired Student’s t-test (GraphPad Prism v6, GraphPad Software Inc., San Diego, USA). Results were considered significant if p<0.05.

For a full description of all the methods used please see S1 Methods.

## Results

### Influenza infection of lung explants increases macrophage PDL1 expression

**Fig 1:**
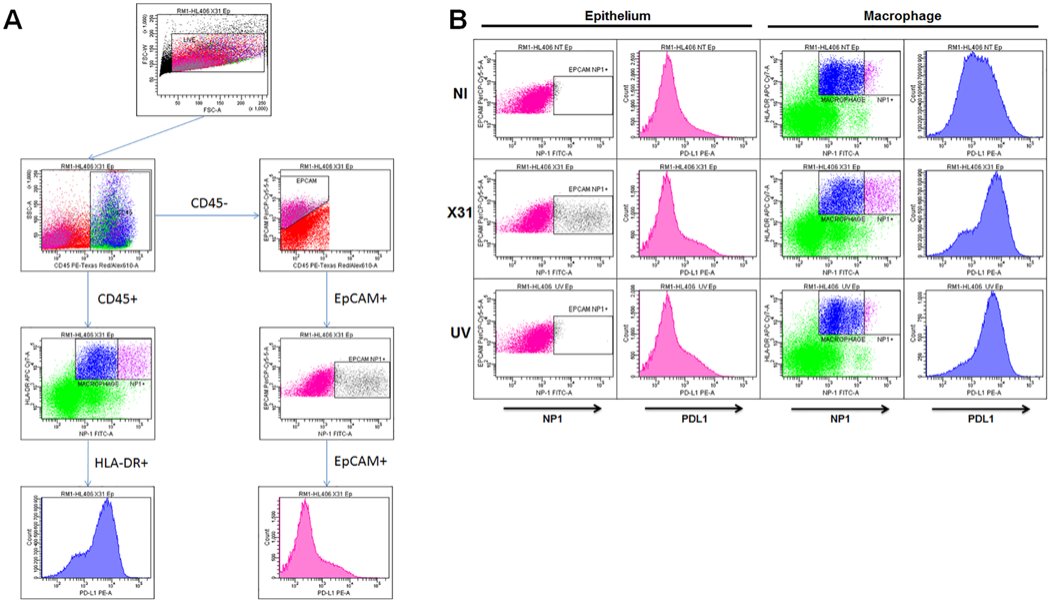
Ex vivo infection of human lung explants. After resting explanted lung tissue overnight, 1 x 10^6^ pfu/ml H3N2 X31 influenza virus or a UV-irradiated aliquot of virus (UVX31) was added for 2 h. After washing, media was replaced and the tissue was incubated for a further 22 h followed by collagenase digestion and flow cytometry analysis **A)** Gating strategy for identification of CD45+HLADR+ macrophages and CD45-EpCAM+ epithelial cells expressing viral NP1 and PDL1 from lung tissue. **B)** FACS plots demonstrating increases in influenza infection (NP1) with corresponding increases in PDL1 expression. Plots are representative of nine independent experiments.

**Fig 2:**
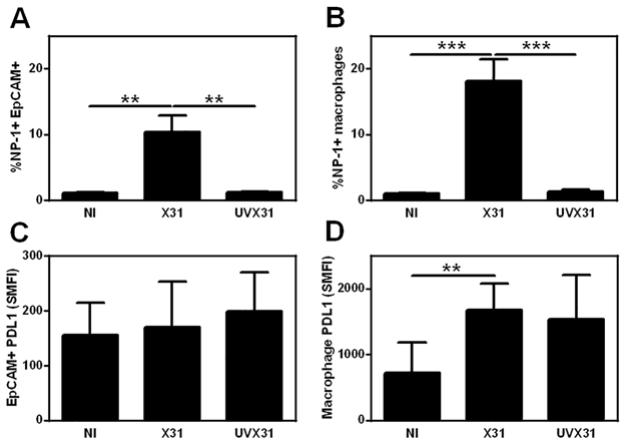
Quantification of infection of human lung explants. Histograms showing flow cytometry data of viral NP1 (% cells) and PDL1 expression (specific mean fluorescence intensity—SMFI) in epithelial cells **(A & C)** and macrophages **(B & D).** Data are expressed as means ±SE of 9 independent experiments. Data analysed using a paired t-test ** p<0.01. **** p<0.0001.

Using the flow cytometry gating strategy we optimised for detection of epithelial cells (EpCAM+) and macrophages (CD45+HLA-DR+—Fig. 1) in the lung explants we quantified the percentage of cells that expressed NP1 in each of these cell types. Approximately 32% of macrophages expressed NP1, as did a mean of 16% of epithelial cells (Fig. 2A & B). This staining was specific to infection and viral replication and not just uptake or binding of the virus to the cell surface, as NP1 staining was not increased above baseline when tissue was incubated with UV-irradiated virus (Fig. 2A & B).

In the same samples we could also analyse the epithelial and macrophage PDL1 expression induced by influenza infection. Prior to infection PDL1 expression was broadly expressed by all the cells in both populations as assessed by comparison to isotype control staining. Macrophages expressed more than double the amount of PDL1 (specific mean fluorescence intensity (SMFI) 255) compared to epithelial cells (SMFI 102). Upon exposure to virus there was no significant increase in epithelial PDL1 expression (Fig. 1B & 2C). In contrast there was a significant increase in PDL1 expression by macrophages infected with X31 and cells exposed to the UV-inactivated virus (Fig. 1B & 2D).

### Isolated lung macrophages also express PD-L1 in response to X31infection

**Fig 3:**
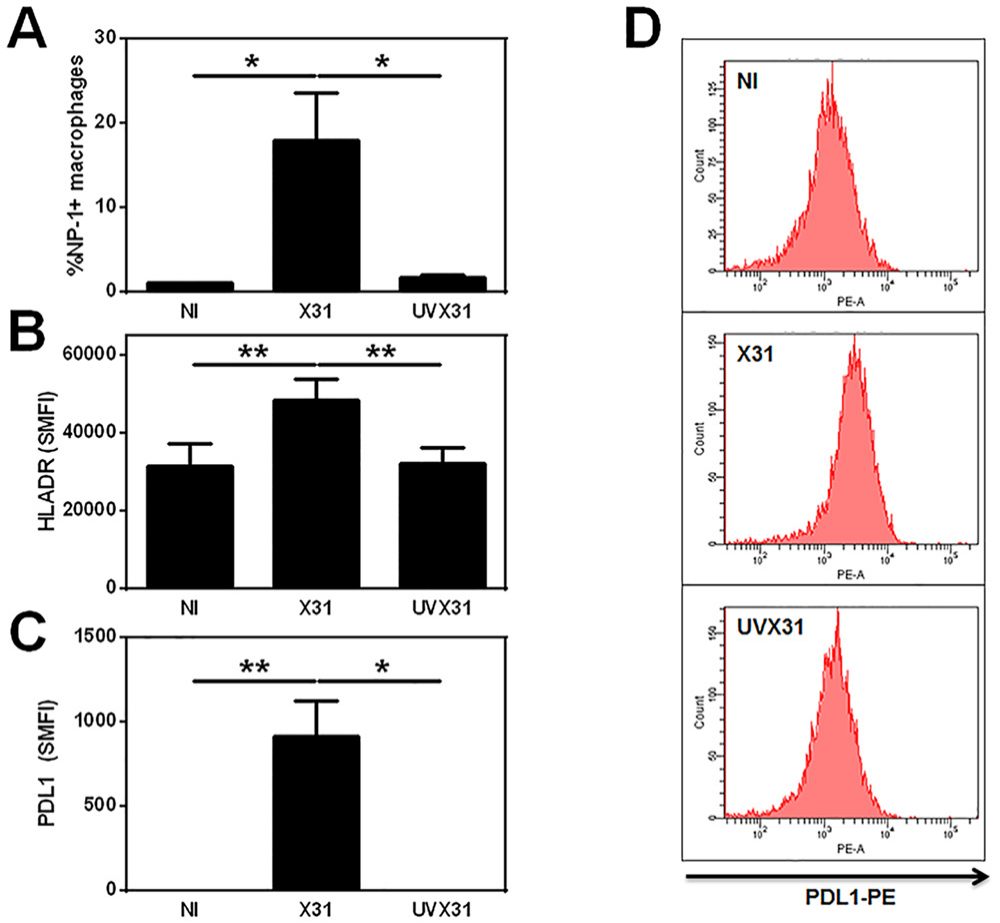
Infection of human lung macrophages by X31. Human lung macrophages were isolated by plate adherence prior to infection with 4000 pfu/ml of H3N2 X31 influenza virus or a UV-irradiated aliquot of virus (UVX31) for 2 h. After washing, media was replaced and the cells incubated for a further 22 h before supernatants and cells were harvested. Cells were analysed for intracellular viral NP1 expression or cell surface expression of HLA-DR and PD-L1 using flow cytometry. Histograms showing flow cytometry data of **A)** Viral NP1 expression (% cells), **B)** HLA-DR expression (specific mean fluorescence intensity—SMFI) and **C)** PDL1 expression (SMFI) from isolated human lung macrophages are expressed as means ±SE of 4 independent experiments. **D)** Representative histograms demonstrating increase in PDL1 expression in response to influenza infection are shown. Data analysed using a paired t-test * p<0.05, ** p<0.01.

To further assess whether this increase in PDL1 expression was due to the tissue milieu or specific to macrophage infection alone, we infected lung macrophages washed out of resected tissue and isolated cells using adherence to plastic. Using non-infected (NI) cells to set a gate for detection of >1% we observed significant numbers of lung macrophages (mean 18%) expressing NP1 compared to both NI and cells exposed to UV-inactivated virus (UVX31—mean 1.7%—Fig. 3A). Infection of the macrophages also caused a significant increase in macrophage activation as assessed by cell surface expression of HLA-DR (Fig. 3B).

In contrast to explant macrophages, prior to infection, isolated lung macrophages expressed little or no PDL1, suggesting that basal PDL1 expression is sustained by the tissue microenvironment. Despite this low basal expression, PDL1 could still be significantly increased in response to live virus but not to UVX31 (Fig. 3C). Expression of PDL1 however was not limited to the cells co-expressing NP1 (Fig. 3D), suggesting that soluble mediators released by the infected cells were responsible for the upregulation of PDL1 on the whole population of macrophages.

### MDM respond to influenza infection similarly to lung macrophages

As the number of macrophages that could be isolated from tissue explants was a potential limiting factor in our study, we generated MDM as previously described[17] to model lung macrophages and assessed whether MDM responded similarly to tissue-derived cells. In initial experiments, the concentration of virus used was titrated to minimise virus-induced toxicity as measured by LDH release into the supernatant and to give similar levels of infection seen in tissue macrophages (see S1 Fig.).

**Fig 4:**
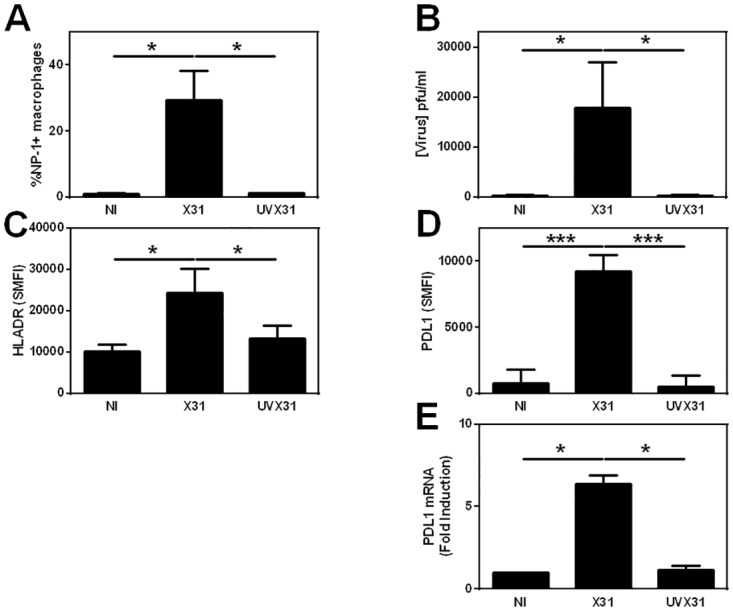
Infection of MDMs by X31. MDM were differentiated in the presence of 2 ng/ml GM-CSF for 12 d prior to infection with 500 pfu/ml of H3N2 X31 influenza virus or a UV-irradiated aliquot of virus (UVX31) for 2 h. After washing, media was replaced and the cells incubated for a further 22 h before supernatants and cells were harvested. Cells were analysed using flow cytometry and RT-PCR, supernatant was analysed using dot blot. Histograms showing infected MDM expression of **A)** Viral NP1 expression (% cells n = 7), **B)** release of hemagglutinin into supernatants (n = 10) **C)** cell surface HLA-DR expression (specific mean fluorescence intensity—SMFI n = 7) **D)**, PDL1 expression (SMFI n = 7) **E)** PDL1 gene expression (n = 6). Data are expressed as means ± SE of n independent experiments and analysed using a paired t-test * p<0.05, ** p<0.01. *** p<0.001.

Using flow cytometry, we detected significant amounts of NP1 expression in X31-exposed MDM after 22 h, but not from UVX31- exposed cells (Fig. 4A). At the same time, we observed significant HA release into the culture media from X31-infected cells after 22 h compared to MDM infected for 2 h, but not from cells treated with UVX31 (Fig. 4B). As the dose of virus used was associated with minimal cell death (S1B Fig.), these data suggest that not only is viral replication occurring in MDM but that new virus is being shed from the surface of these cells.

Similarly to explant macrophages, we observed low basal levels of PDL1 on the MDM cell surface and this expression was significantly increased in response to infection with X31 but not UV-inactivated virus (Fig. 4D). Expression of the MHC class II molecule, HLA-DR, was also significantly increased upon infection of MDM with X31 as was CD86 (Fig. 4C and S1C Fig.), suggesting activation and upregulation of antigen-presentation pathways in these cells. In contrast, the expression of CD14 and PDL2 was not significantly altered in response to viral infection (S1D & E Fig.).

In order to discover if influenza infection increased PDL1 surface expression on MDM by activating PDL1 gene expression, we analysed RNA isolated from MDM infected with UV-inactivated or live virus for 24 h by RT-PCR. Viral infection caused a significant 6.4-fold increase in the steady-state levels of PDL1 mRNA when compared to uninfected cells and exposure to UVX31 did not lead to an increase in expression of this gene (Fig. 4E). Moreover, this effect was not limited to influenza, as infecting the MDM with respiratory syncytial virus (RSV) also increased PDL1 expression (S2 Fig.).

### Functional consequence of virus-induced PDL1 expression

**Fig 5:**
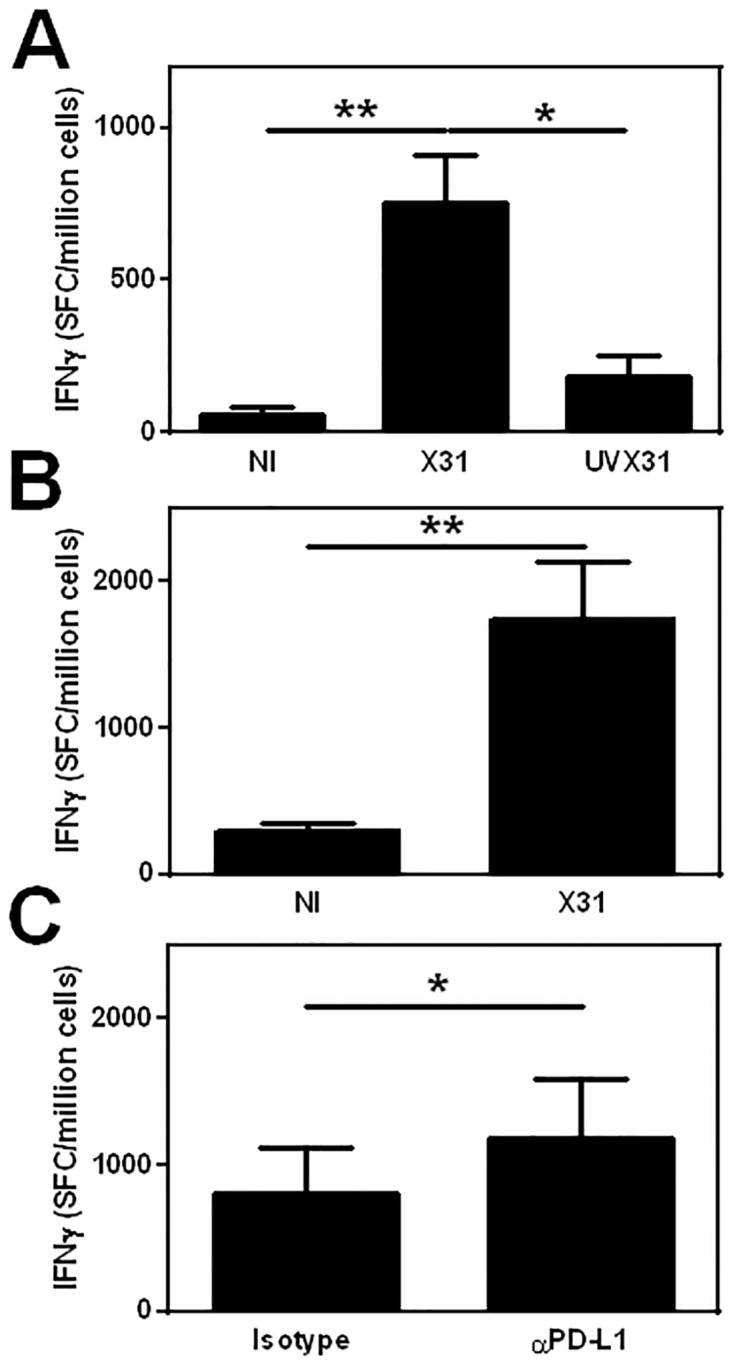
Functional effect of PDL1 on T cell activation. MDM were differentiated in the presence of 2 ng/ml GM-CSF for 12 d prior to infection with 500 pfu/ml of H3N2 X31 influenza virus or a UV-irradiated aliquot of virus (UVX31) for 2 h. Infected MDM were transferred to a coated ELISpot plate and autologous lymphocytes added and incubated for a further 22 h before IFNγ release was measured using ELISpot. **A)** 2.5 x 10^5^ monocyte-depleted PBMC co-cultured with 5 x 10^4^ autologous MDM not infected (NI) or treated with X31 or UVX31(n = 5). **B)** 1 x 10^5^ CD8+ T cells co-cultured with 5 x 10^4^ autologous MDM NI or infected with X31 (n = 6) **C)** 1 x 10^5^ CD8+ T cells co-cultured with 5 x 10^4^ autologous MDM infected with X31 in the presence of 10 μg/ml anti-PDL1 antibody or isotype control (n = 5). Data are expressed as means ±SE of n independent experiments and analysed using a paired t-test * p<0.05, ** p<0.01.

To assess the functional significance of this virus-induced PDL1 expression by macrophages, we co-cultured autologous lymphocytes with influenza infected macrophages and analysed IFNγ release using ELISpot (Fig. 5). X31-infected MDM elicited significantly greater IFNγ release than uninfected or UVX31-exposed cells (Fig. 5A) although there was a trend for greater IFNγ release from autologous lymphocytes co-incubated with UVX31-exposed cells compared to uninfected cultures (p = 0.0659). We extended these investigations by incubating the infected macrophages with autologous CD8+ T cells and again observed a significant increase in the amount of IFNγ detected (Fig. 5B). Moreover, the amount of IFNγ released by autologous T cells was significantly enhanced when the infected MDM-CD8+ cell cultures from every donor were incubated in the presence of a PDL1 blocking antibody (10 μg/ml), when compared to incubating with an isotype control antibody (Fig. 5C). These data suggest that the PDL1 on the macrophage surface is negatively regulating CD8+ T cell activation in line with previous investigations [7].

### Influenza infection induces pro-inflammatory cytokine expression in MDM

The observation that increased macrophage PDL1 expression was not limited solely to NP1+ cells, suggested that soluble factors released by the infected cells were responsible for the up-regulation of PDL1. Since the release and expression of interferons (IFNs) is the archetypal response to viral infection we therefore investigated the expression of these cytokines by MDM infected with X31.

**Fig 6:**
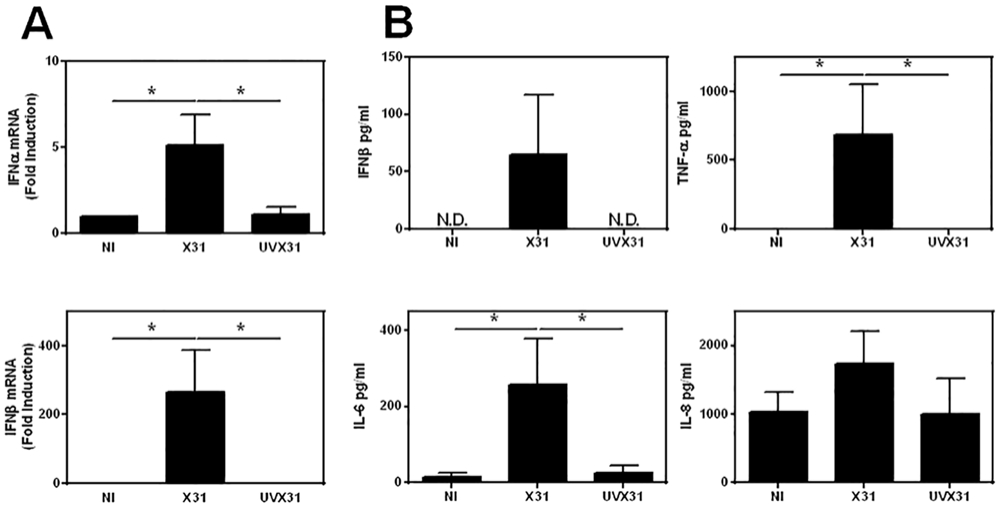
Effects of influenza infection on MDM cytokine expression. MDM were differentiated in the presence of 2 ng/ml GM-CSF for 12 d prior to infection with 500 pfu/ml of H3N2 X31 influenza virus or a UV-irradiated aliquot of virus (UVX31) for 2 h. After washing, media was replaced and the cells incubated for a further 22 h before supernatants and cells were harvested. Inflammatory cytokine expression was measured by **A)** real time PCR (IFNα, IFNβ n = 6) and **B)** ELISA (IFNβ, TNFα, IL-6, IL-8 n = 6) N.D. = not detected. PCR data were normalised to β2MG and are expressed as mean fold induction over the non-infected (NI) sample ± SEM of 6 independent experiments. ELISA data are expressed as means ±SE of 6 independent experiments. Data analysed using a paired t-test. * p<0.05, ** p<0.01.

MDM were exposed to virus for 2 h, washed and lysed immediately or media replaced and incubated for a further 22 h before cell lysis. IFNγ gene expression was below the limit of detection (>40 cycles) at both time points in these experiments and was not detectable in any of the MDM culture supernatants in line with our ELISpot data (data not shown). In contrast IFNβ, was elevated after infection with X31 for 2 h (data not shown), and this expression was significantly increased after 24 h infection (Fig. 6A). Relative induction of IFNα was lower than IFNβ but followed a similar pattern with significant expression after infection for 24 h. No IFNβ could be detected in any supernatants from uninfected or UVX31 exposed cells, but detectable levels of IFNβ were released into the MDM culture supernatants in response to 24 h infection with X31 (64.7 ± 52.5 pg/ml n = 6—Fig. 6B). Furthermore, infecting MDM with RSV also caused a significant upregulation of IFNβ gene expression underlining the importance of this cytokine in antiviral responses (Supporting Information Fig. 2B).

Significant increases in TNF and IL-6, but not IL-8 were also detected in supernatants from X31 infected MDMs when compared to uninfected cells (Fig. 6B). To exclude a role for TNF and IL-6 in the upregulation of PDL1 by macrophages we incubated MDM with TNF and IL-6 (both 10 ng/ml) for 24 h. TNF only induced a modest 1.5-fold increase in PDL1 surface expression, whilst IL-6 (10 ng/ml) had no effect on PDL1 expression (both n = 3—data not shown).

### Role of virus-induced IFNβ in PDL1 expression

**Fig 7:**
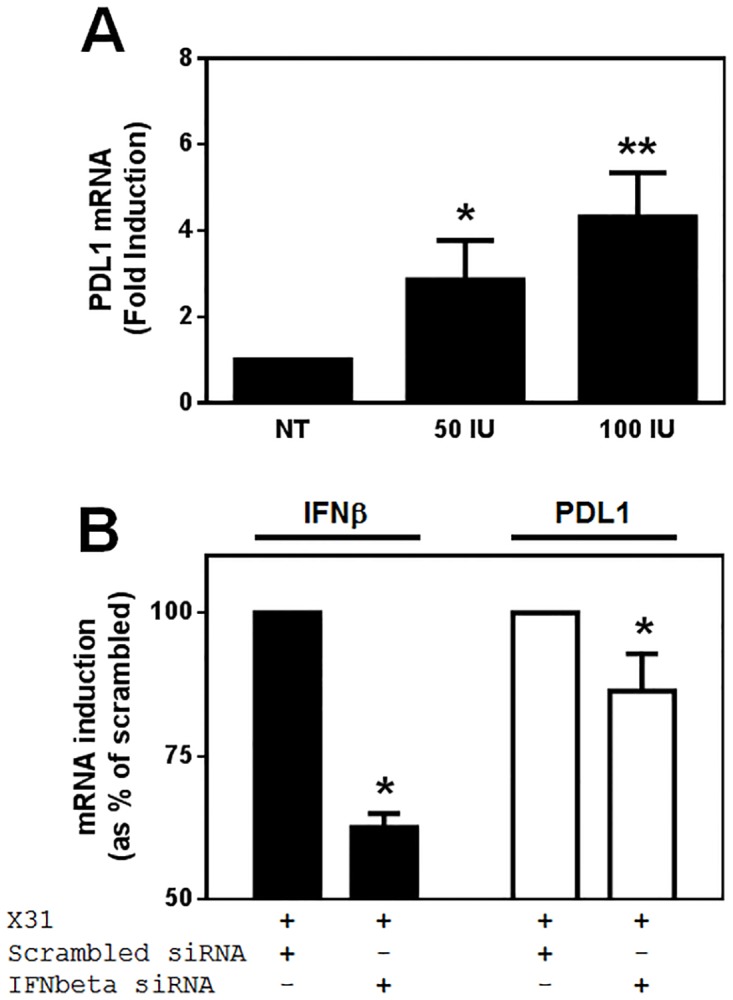
Regulation of PDL1 mRNA expression by influenza infection and IFNβ. **A)** PDL1 gene expression was assessed in MDM differentiated in the presence of 2 ng/ml GM-CSF for 12 d before incubation with the indicated doses of rhIFNβ for 24 h using RT-PCR (n = 5). **B)** MDM were differentiated in the presence of 2 ng/ml GM-CSF for 11 d before transfection with non-specific siRNA or siRNA specific for IFNβ in the presence of GM-CSF for 24 h. MDM were then infected with 500 pfu/ml of H3N2 X31 influenza virus or a UV-irradiated aliquot of virus (UVX31) for 2 h. After washing, media was replaced and the cells incubated for a further 22 h before cells were harvested for RT-PCR analysis of IFNβ and PDL1 mRNA expression (n = 6). PCR data were normalised to β2MG and are expressed as mean fold induction over the non-infected (NI) sample ± SEM of n independent experiments. Data analysed using a paired t-test * p<0.05, ** p<0.01.

Since type I IFN expression was observed in response to influenza infection and previous studies have suggested that these cytokines could induce PDL1 expression[18,19], we investigated whether rhIFNβ could induce PDL1 gene expression in our MDM model (Fig. 7A). We observed a dose dependent-increase in PDL1 steady-state mRNA expression in response to IFNβ after 24 h. Using 50 IU of rhIFNβ we saw a 2.9-fold increase in PDL1 gene expression increasing to 4.3-fold at 100 IU of this cytokine.

We confirmed that influenza infection induces PDL1 via release of IFNβ by using short interfering (si)RNA directed against IFNβ. After isolation, monocytes were cultured in GM-CSF media for 11 d before incubating with scrambled siRNA or siRNA specific to IFNβ for 24 h in GM-CSF media. MDM were infected with influenza as previously described and IFNβ and PDL1 mRNA expression were analysed using RT-PCR. Compared to the scrambled siRNA, the specific RNA significantly inhibited IFNβ mRNA expression by 37% (Fig. 7B). However despite this limited inhibition of IFNβ message, PDL1 mRNA was also significantly inhibited by siRNA targeted against IFNβ, albeit to a lesser degree (14% inhibition).

## Discussion

In the present study, we have demonstrated for the first time that in the acute phase of influenza infection macrophages are the primary cells in the human lung expressing PDL1. This macrophage PDL1 expression has functional effects on autologous co-cultured CD8+ T cells. Furthermore, these changes in PDL1 expression were not limited to NP1+ cells and hence appear to be mediated by release of IFNβ. Whilst the expression of PDL1 by human macrophages has been investigated in response to both HIV and HCV infection, to our knowledge this is the first investigation into the mechanisms of regulation of this molecule in human macrophages infected with respiratory viruses, such as RSV and influenza.

Influenza infection of the airway is known to affect the macrophage functions of phagocytosis and bacterial killing[20], but the question as to whether this was mediated by exogenous factors or by infection of the macrophages themselves has remained open. Early studies demonstrated that virus-induced suppression of antibacterial defences was mediated by lymphocytes[21]. Subsequent studies have suggested that it is the cytokines released by lymphocytes, such as IFNγ and IL-10 that mediate the suppressive effects of virus[1,22–24]. However, as influenza can infect and replicate in human alveolar macrophages[1], the reciprocal effects of influenza-infected macrophages on lymphocyte function have remained largely unexplored. The recent study by Erickson et al (2012) demonstrating that influenza infection of the lung can upregulate PDL1 expression on both the airway epithelium and macrophages and that this upregulation directly reduces CD8 T cell effector function highlights the dynamic relationship between infected macrophages and T cells[7]. In contrast to this previous study we did not observe an up-regulation in epithelial PDL1 expression in response to influenza infection. However, Erickson et al (2012) analysed post-mortem lung samples from individuals with severe influenza infection and thus the epithelial PDL1 expression observed may be at a more advanced stage of infection than our acute infection model can reflect[7]. Furthermore, our data suggest the importance of macrophage PDL1 expression controlling T cell responses earlier on in infection.

Expression of the T cell stimulatory molecules, HLA-DR and CD86, as well as the inhibitory PDL1 protein was induced on influenza-infected macrophages. HLA-DR is a component of the Major Histocompatibility Class (MHC) II complex involved in antigen presentation to CD4+ T cells[25], whilst CD86 is classically associated with activation of the CD28 signalling pathway[26]. Why the infected macrophage should upregulate both stimulatory and inhibitory signals at the same time and the functional significance of this is open for speculation. Previous investigators have suggested that there is equilibrium between expression of these markers and the activation state, with inhibitory signalling being predominant when CD80/CD86 expression is low[27]. Selenko-Gebauer et al (2003) hypothesised that PDL1 expression may function as an immunological rheostat to set a T cell activation threshold[27]. One further possibility is suggested by the fact that both CD80 and CD86 can induce signalling by the inhibitory CTLA-4 receptor on T cells as well as activating the CD28 pathway[28], therefore an inhibitory milieu may be propagated via the infected macrophage. This may be especially important in the lung as unconstrained inflammation can lead to lung function impairment and death[6].

Both type I and type II IFNs have been demonstrated to increase macrophage PDL1 expression[9]. We observed a trend towards increased blood T cell IFNγ release in response to the UV-inactivated virus and it is likely that there are more virus-specific memory T cells in the lung than in the blood. The release of IFNγ from lung resident memory T cells may therefore explain the difference in PDL1 expression between tissue macrophages and isolated macrophages exposed to UVX31. However, as IFNγ was not expressed by isolated macrophages in response to infection we focused on the role of type I IFNs in PDL1 expression in isolated cells. Levels of IFNβ released from uninfected MDM were below the limit of detection, whereas influenza infection caused MDM to release measurable quantities of IFNβ and addition of as little as 50 IU of this cytokine caused an increase in PDL1 mRNA expression. This amount of IFNβ corresponds to approximately 250 pg of IFNβ which is within the same order of magnitude as that detected from our infected-MDM by ELISA. Furthermore, we have used siRNA targeted against IFNβ to demonstrate a role for this cytokine in the influenza-induced expression of PDL1. Significant attempts to optimise the efficiency of the knockdown by varying the amounts of siRNA and transfection reagent and the time of siRNA addition, but we were unable to improve the knockdown beyond a maximum of approximately 45%. It is possible that knockdown could be improved further by reducing the number of cells to be transfected but in this investigation we had to strike a balance between efficient siRNA transfection and optimized influenza infection. We therefore opted to retain the conditions optimal for influenza infection. Despite the moderate knockdown of IFNβ mRNA induced by the specific siRNA, PDL1 could still be significantly inhibited. This effect seems to be specific to IFNβ as in preliminary experiments we did not observe any effect of IFNα knockdown on PDL1 expression (data not shown). These observations are in line with previous work showing a significant increase in PDL1 expression on blood monocytes in multiple sclerosis patients undergoing IFNβ therapy[19].

The data presented herein may have implications for understanding the mechanisms of the virus-induced exacerbations of chronic respiratory diseases, such as severe asthma[29]. Asthma has long been associated with a skewing of lung immunity away from a Th1 (i.e. IFNγ producing) response to a high Th2 (i.e. IL-4/13 producing) response[30] and more recent work has demonstrated no significant induction of IFNγ in BAL cells from asthma patients experimentally challenged with rhinovirus[31]. Additionally, previous work has demonstrated a defect in both epithelial[32,33] and bronchoalveolar lavage cell[29] production of type I and type III IFNs in asthma in response to viral infection. Thus, in addition to the increase of viral replication resulting from decreased IFN production, the worsening of symptoms and increased inflammatory burden associated with asthma exacerbations may also be enhanced by decreased IFN-induced PDL1 expression failing to adequately control the inflammatory response to virus. Further work will be required to fully assess this possibility.

In conclusion, PDL1 expression is a primary response of monocytes/macrophages to viral infection, whether that be in the liver[11], the blood[34] or the lung[7]. Moreover the role of IFNs in upregulating PDL1 is not confined to monocytes/macrophages as IFNα and IFNγ have both been shown to increase expression of PDL1 in hepatocytes[35]. Taken together with our data using influenza and RSV, we suggest that this increased PDL1 expression is a common host response to infection by different virus families. The question remains as to why this host response mechanism in response to virus has developed and what its functional significance is? Perhaps it is a means by which anti-viral responses can be restrained before the resulting inflammation can cause too much tissue damage, as has been suggested in the liver[36]. Or, as has been suggested for HIV, this mechanism has been subverted by the virus to prevent viral clearance[37]. In terms of influenza, PDL1 co-inhibition of virus-specific memory T cells may explain the partial responses to immunization and poor cross protection from one influenza season to the next.

## Supporting Information

S1 Methods A full description of all the methods used to generate the data contained within this paper. (DOC)

S1 Fig Viability and cell surface marker expression of infected MDM. MDM were infected with increasing concentrations of X31 virus for 24 h and A) cells were analysed for viral NP-1 expression using flow cytometry and the percentage of NP1+ cells were gated using uninfected cells as a negative control. B) supernatants were harvested for LDH analysis. Data are expressed as means ± SE of 3 independent experiments. C—E) Histograms showing infected MDM expression of cell surface C) CD86 expression (specific mean fluorescence intensity—SMFI n = 4) and B) PDL2 expression (SMFI n = 7) E) CD14 expression (SMFI n = 4) expressed as means ± SE of n independent experiments. ** P<0.01, *** P<0.001
(PPTX)

S2 Fig Infection of MDM by RSV. After infection with RSV, MDM were lysed and expression of A) RSV-N gene at 2 h and 24 h post infection, B) IFNβ gene expression at 24 h and C) PDL1 gene expression were measured by RT-PCR and normalized to b2-microglobulin. Data are expressed as mean ± SE 2^-delta Ct values of 7 independent experiments. * P<0.05, ** P<0.01.
(PPTX)

S3 Fig Analysis of sensitivity of lymphocytes to X31 infection. **A)** MDMs or lymphocytes were treated with UVX31 or X31 for 2 h before washing. 5 x 10^4^ MDMs were added to wells of an ELISPOT plate and were co-cultured with 2.5 x 10^5^ lymphocytes. Wells not containing MDMs (T only), and wells containing MDMs only were also measured for their ability to produce IFNγ. The ELISPOT plate was incubated at 37°C for 18 h before analysis. Mean and SEM shown of 3 independent experiments. Paired students t test was performed. p < 0.05 (*). **B)** Graph showing mean percentage (± SE n = 3 independent experiments) infection of CD14^+^ monocytes, CD4^+^ and CD8^+^ T lymphocytes fractions of PBMC cultures exposed to increasing concentrations of X31 influenza virus for 20 h as assessed by flow cytometry. **C)** Representative FACS plots of 3 independent experiments at the highest dose of virus used are shown.
(PPTX)
