# Supplementary material for: Viral Infection of Human Lung Macrophages Increases PDL1 Expression via IFNβ
Source: PLoS One. 2015 Mar 16;10(3):e0121527. doi: 10.1371/journal.pone.0121527 (PMC4361055; doi:10.1371/journal.pone.0121527)
Supplement: S1 Methods — (DOC) [file pone.0121527.s001.doc]

**Supporting Information Methods for:**

**Viral infection of human lung macrophages increases PDL1 expression via IFNβ.**

Karl J. Staples, Ph.D.^1,3^, Ben Nicholas, Ph.D.^1^, Richard T. McKendry, B.Sc.(Hons)^1^, C. Mirella Spalluto, Ph.D.^1^, Joshua C. Wallington, B.Sc.(Hons)^1^, Craig W. Bragg, B.Sc.(Hons)^1^, Emily C. Robinson, B.Sc.(Hons)^1^, Kirstin Martin, BM^1^, Ratko Djukanović, MD^1,2^, Tom MA Wilkinson, Ph.D.^1^.

**Methods**

**Ex vivo infection of resected human lung tissue**

Lung tissue was obtained from patients undergoing airway re-sectioning surgery at Southampton General Hospital. The collection of tissues was approved by and performed in accordance with the ethical standards of the Southampton and South West Hampshire Research Ethics Committee, LREC no: 09/H0504/109. Parenchymal tissue, distant from the resection margin and any gross pathology was dissected from the lobe. Tissue was cut into 1mm^3^ sections and added to a 24-well flat-bottomed culture plate (Sigma, Poole, UK) before washing with DPBS. Washing of the tissue was performed by removing DPBS from the wells and replacing it with fresh DPBS, followed by unsupplemented RPMI and finally RPMI supplemented with 1% penicillin/streptomycin (Gibco Life Sciences) and 1% gentamycin (PAA). Tissue was then incubated overnight at 37ºC and 5% CO_2_. The wet weight of tissue was recorded.

Tissue was infected and analysed according to according to the method described by Nicholas et al (2012). After resting overnight the media was replaced with serum-free RPMI supplemented with 100 U/ml penicillin, 100 µg/ml streptomycin, 2 mM L-glutamine and 250 ng/ml fungizone. Influenza A virus strain X31 was supplied at a concentration of 4 x 10^7^ pfu/ml (a kind gift of 3VBiosciences). Inactivated virus (UVX31) was prepared by exposure of the X31 to an ultra-violet (UV) light source for 2 h. X31 or UVX31 at a concentration of 1 x 10^6^ pfu/ml was added to designated wells, with control non-infected wells. Tissue was incubated at 37ºC and 5% CO_2_ for 2h to allow for infection of cells residing in the tissue. Supernatant was removed and tissue was washed three times with unsupplemented RPMI in order to remove excess virus from the wells. Serum-free RPMI supplemented with 100 U/ml penicillin, 100µg/ml streptomycin, 2 mM L-glutamine and 250 ng/ml fungizone was added to the wells and tissue was incubated at 37ºC and 5% CO_2_ for a further 22 h.

Protocol used to digest tissue was adapted from Holt *et al* . Briefly, Tissue was added to a solution of pre-warmed unsupplemented RPMI and 1 mg/ml collagenase type I (Sigma, Poole, UK) for digestion. A magnetic stirrer was added to the solution in order to mechanically disaggregate tissue. Collagenase digestion occurred at 37ºC for 15 min. After digestion the solution was filtered through a 35 µm pore straining cap into 5 ml round-bottomed polypropylene FACS tubes (BD Biosciences) in preparation for FACS analysis.

**Lung macrophage isolation from resected human lung tissue**

Resected human lung tissue was obtained from patients as described above and macrophages were isolated according to previously published protocols. Briefly, tissue was washed extensively with basal RPMI and the wash solution was then centrifuged to pellet any cells and the red blood cells were lysed with a hypotonic solution. Following centrifugation, the cell pellet was resuspended in serum-free RPMI supplemented with 2 mg/ml L-Glutamine, 0.05 U/ml penicillin, 50 µg/ml streptomycin, 0.5 mg/ml amphotericin B (all Invitrogen, Paisley, UK) and transferred to a cell culture dish. After 2 h adherence on plastic, non-adherent cells were washed off and the remaining cells were cultured overnight in complete RPMI, supplemented as above but with the addition of 10% heat-inactivated fetal bovine serum (FBS - Invitrogen). Preliminary experiments demonstrated that cells isolated in this way were >95% macrophages using DC-SIGN expression (data not shown). Macrophages were then washed extensively with basal RPMI before addition of virus in reduced serum (RS – supplemented as above except 0.1% FBS) RPMI. Collection of tissue for this part of the study was approved by the Southampton and South West Hampshire Research Ethics Committee (reference: 09/H0504/109).

**Monocyte Isolation & differentiation**

Human peripheral blood mononuclear cells (PBMC) were isolated from heparinised blood by centrifugation on Lymphoprep® (Axis-Shield, Oslo, Norway). Monocytes were then isolated from the PBMC using CD14+ microbeads (Miltenyi-Biotec, Bisley, UK) according to the manufacturer’s instructions. Isolated monocytes were resuspended in complete RPMI supplemented with 2 ng/ml GM-CSF (R&D Systems, Abingdon, UK). MDM were then washed extensively with basal RPMI before addition of virus, rhIL-10 (R&D) or rhIFNβ (National Institute for Biological Standards and Control, Potters Bar, UK) in RS-RPMI. Collection of samples for this part of the study was approved by the Southampton and South West Hampshire Research Ethics Committee (reference: 08/H0504/138). Monocyte-depleted PBMC that resulted from this separation step were frozen at -80°C in 10% (v/v) DMSO/HI-FBS for use in later ELISpot analysis.

**Infection of lung macrophages and MDM**

Influenza A virus strain X31 was supplied at a concentration of 4 x 10^7^ pfu/ml (a kind gift of 3VBiosciences). Inactivated virus (UVX31) was prepared by exposure of the X31 to an ultra-violet (UV) light source for 2 h. Macrophages were incubated for 2 h with no virus, or 4000 pfu (lung) or 500 pfu (MDM) of X31 or UVX31. Supernatants were harvested (T-2), the cells washed three times, the final wash was harvested (T0) and fresh RS media was added to the MDMs. Cells were then washed and incubated for a further 22 h at 37°C, 5% CO_2_. After a further 22 h, supernatants were harvested (T22) for HA shedding and LDH assays and cells collected and immediately analysed by flow cytometry. For phenotypic characterisation of influenza infected macrophages, cells were removed from culture plates using a non-enzymatic cell dissociation solution (Sigma, Poole, UK).

A similar method was used to infect MDM with Respiratory Syncytial Virus (RSV - strain M37 - Meridian Life Science Inc, Memphis, USA). Inactivated RSV (UV-RSV) was prepared by exposure of the X31 to an ultra-violet (UV) light source for 2 h. 500 µl of stock RSV (3.5 x 10^6^ pfu) was diluted 1:1 in basal RPMI; and a 1:10 dilution was made in each well and incubated for 2 h at 37°C. MDMs were then washed with basal RPMI and cultured in RS RPMI for a further 22 h.

**Flow cytometry analysis**

Samples were resuspended in FACS buffer (PBS, 0.5% w/v BSA, 2 mM EDTA) containing 2 mg/ml human IgG

Lung explants:

Single cell suspensions derived from collagenase digestion were incubated on ice in the dark for 30 min with the following antibodies: Phycoerythrin-CF594 (PE-CF-594)-conjugated anti-CD45, Peridinin-Chlorophyll Protein-Cyanine 5.5 (PerCPCy-5.5)-conjugated anti-EpCAM-1 (CD326), and Allophycocyanin-Cyanine 7 (APC-Cy7)-conjugated anti-HLA-DR, Phycoerythrin (PE)-conjugated anti-PD-L1 (All BD Biosciences, Oxford, UK). Appropriate isotype and fluorescence-matched control antibodies were added in a sample of the cells to aid gating of cell populations. After washing, intracellular staining for viral nucleoprotein (NP)-1, was performed using BD Cytofix/Cytoperm kit according to manufacturer’s instructions, and AlexaFluor 488 (AF488)-conjugated anti-NP1 antibody (HB-65, a kind gift of 3VBiosciences). The gating strategy is shown in Figure 1A.

Lung macrophages and MDMs:

Isolated macrophages were incubated on ice in the dark for 30 min with the following antibodies: APC-Cy7-conjugated anti-HLA-DR, PE-conjugated anti-PD-L1, Allophycocyanin (APC)-conjugated anti-CD86, PerCPCy-5.5-conjugated anti CD14 (All BD Biosciences), and PE-conjugated anti-PD-L2 (R&D Systems) or appropriate isotype controls. After washing, intracellular staining for viral nucleoprotein (NP)-1, was performed using BD Cytofix/Cytoperm kit according to manufacturer’s instructions, and AlexaFluor 488 (AF488)-conjugated anti-NP1 antibody (HB-65, a kind gift of 3VBiosciences).

Flow cytometric analysis was performed on a FACSAria using FACSDiva software v5.0.3 (all BD).

**ELISpot**

Monocyte-depleted PBMC were defrosted at room temperature and suspended in RS RPMI. MDMs were removed from 24-well culture plate using non-enzymatic cell dissociation solution (Sigma) and transferred to sterile 1.5 ml Eppendorf tubes. Cells were centrifuged at 400 g, 4ºC, 5 min before resuspension in serum-free (SF) RPMI containing L-glutamine, penicillin/streptomycin, and fungizone. MDMs were either not infected, or were treated with 2.5 x 10^4^ pfu/ml X31 Influenza A H3N2 virus at 37ºC for 2 h before washing and resuspending in RS RPMI.

ELISpot for Human IFN-γ was then performed using 0.45 µm MultiScreen-IP Filter Plates (Millipore, Watford, UK) following manufacturer’s instructions (MabTech, Stockholm, Sweden). Briefly, coating antibody (1-DIK) was diluted to 15 µg/ml in sterile DPBS and was added to the plate before overnight incubation at 4ºC. Plates were then washed five times with sterile DPBS before replacement with SF RPMI for 30 min at RT. The SF-media was then removed and MDM were added to each well at a concentration of 5 x 10^4^ cells/well and 2.5 x 10^5^ monocyte-depleted PBMC or 1 x 10^5^ CD8+ T cells were added to MDM-containing wells and incubated at 37ºC. After 22 h, the plate was washed five times with sterile DPBS + 0.05% Tween20 (Sigma). Detection antibody (7-B6-biotin) was diluted to 1 µg/ml in sterile DPBS + 0.5% FBS and was added to the plate which was then incubated at RT. After 2 h, the plate was washed five times with sterile DPBS before addition of Streptavidin-ALP (diluted 1:1000 in sterile DPBS+0.5% FCS) and incubation for 1 h at RT. Plates were then washed with sterile DPBS before replacement with substrate solution (BCIP/NBT diluted 1:1:8 in sterile H_2_O). Plate was incubated at RT for 2-5m until clear spots were visible. At this point wells were washed five times with dH_2_O and allowed to dry at RT. Spot development was analysed using an AID EliSpot Reader (Germany) and AID EliSpot Software (Germany).

In initial experiments, no IFNγ staining was seen in wells containing infected MDM or lymphocytes alone (Supporting Information Fig S3A). Peripheral blood T cells do not appear to be infected when exposed to X31 (Supporting Information Fig S3B).

**Modulation of IFNβ mRNA using siRNA**

After isolation, monocytes were cultured in GM-CSF media as described above for 11 d before cells were washed with basal RPMI. One hundred microliters of GM-CSF media was added to the cultures before incubating with 50 nM scrambled siRNA or siRNA specific to IFNβ using 6 µl HiPerFect (Qiagen, Manchester, UK) for 24 h in GM-CSF media. MDM were subsequently infected with influenza as previously described and IFNβ and PD-L1 mRNA expression were analysed using RT-PCR.

**RNA Isolation & RT-PCR**

Lysis buffer was added directly to cells after removal of supernatants. Lysis buffer was then removed to eppendorf tubes prior to storage at -80°C. RNA was isolated using a Stratagene Microprep Kit (Amsterdam, NL). Reverse transcription was carried out using a PrimerDesign nanoScript Reverse Transcriptase kit (Southampton, UK). PCR amplifications were performed on a BioRad iCycler (Hemel Hempstead, UK) using PrimerDesign Precision 2X qPCR Mastermix with or without SYBR Green as appropriate and the following primers (all PrimerDesign Ltd):

IFNα: Fwd 5′- CTA GAG CCC AAG GTT CAG AGT -3′.

Rev 5′- CAC CAC CAG GAC CAT CAG TA -3′.

IFNβ: Fwd 5′-TTA CTT CAT TAA CAG ACT TAC AGG T -3′.

Rev 5′- TAC ATT AGC CAT CAG TCA CTT AAA C -3′.

IFNγ: Fwd 5′- TCA ACT TCT TTG GCT TAA TTC TCT C -3′.

Rev 5′- ATA TGG GTC CTG GCA GTA ACA -3′.

IL-10: Fwd 5′- GCT GGA GGA CTT TAA GGG TTA C -3′.

Rev 5′- TGA TGT CTG GGT CTT GGT TCT -3′.

TGFβ: Fwd 5′- CAC TCC CAC TCC CTC TCT C -3′.

Rev 5′- GTC CCC TGT GCC TTG ATG -3′.

FAM-labelled PDL1 primers (Hs01125301_m1) were obtained from Applied Biosystems. Gene expression was normalized to the relative concentration of the housekeeping gene, β_2_ microglobulin (β2MG – designed and validated by PrimerDesign Ltd), determined in a separate PCR.

**Supernatant analyses**

IFNβ concentrations in culture supernatants were measured by ELISA according to the manufacturer’s instructions (MSD, Gaithersberg, USA). Culture supernatants were analysed by Luminex assay for IL-2, IL-4, IL-5, IL-6, IL-8, IL-10, IL-12p70, IL-13, TNFα, and IFNγ as per manufacturer’s instructions (Bio-Rad). LDH release was measured using CytoTox 96® Non-Radioactivity Cytotoxicity Assay according to the manufacturer’s instructions (Promega, Southampton, UK). Release of viral hemagglutinin was measured using a dot blot assay. Briefly, culture supernatants were diluted 1:5 in PBS and transferred to pre-wetted nitrocellulose membrane in a 96-well array format using BioRad biodot apparatus. Standard curves were generated using purified virus preparations diluted in culture medium. Membranes were then blocked in 5% (w/v) non-fat dried milk powder in PBS-0.05% (v/v) Tween 20 for 1 h at room temperature. Viral proteins were detected using a rabbit polyclonal anti-influenza serum diluted 1/5,000 in blocking solution) overnight at 4^o^C. The bound rabbit antibodies were detected using the Bio-Rad anti-Rabbit HRP detection system according to the manufacturer’s instructions, where the secondary antibody (goat anti-rabbit HRP conjugate) was diluted 1/10,000 in PBS-Tween and incubated with the membrane for 2 h. Chemiluminscence images were captured using a Versadoc imager, and dot array quantities (OD/mm^2^) were analysed using QuantityOne software (BioRad, Hercules, Ca, USA). Specific viral release into the culture medium was analysed from the standard curve using sample values (pfu/ml) from which the background (post-infection) virus concentrations had been subtracted.

**Statistics**

Statistical analyses were performed using a paired Student’s t-test (GraphPad Prism v6, GraphPad Software Inc., San Diego, USA). Results were considered significant if p<0.05.

**References**

S1. Nicholas B, Staples K, Moese S, Ward J, North M, et al. (2012) Validation Of Anti-Host Cell Influenza Targets Using A Novel Human Lung Tissue Model. Am J Respir Crit Care Med 185: A5716.

S2. Holt PG, Robinson BW, Reid M, Kees UR, Warton A, et al. (1986) Extraction of immune and inflammatory cells from human lung parenchyma: evaluation of an enzymatic digestion procedure. Clin Exp Immunol 66: 188-200.

S3. Sallusto F, Lanzavecchia A (1994) Efficient presentation of soluble antigen by cultured human dendritic cells is maintained by granulocyte/macrophage colony-stimulating factor plus interleukin 4 and downregulated by tumor necrosis factor alpha. J Exp Med 179: 1109-1118.
