# Supplementary figures and images for: Viral Infection of Human Lung Macrophages Increases PDL1 Expression via IFNβ
Source: PLoS One. 2015 Mar 16;10(3):e0121527. doi: 10.1371/journal.pone.0121527 (PMC4361055; doi:10.1371/journal.pone.0121527)

## Slide 1
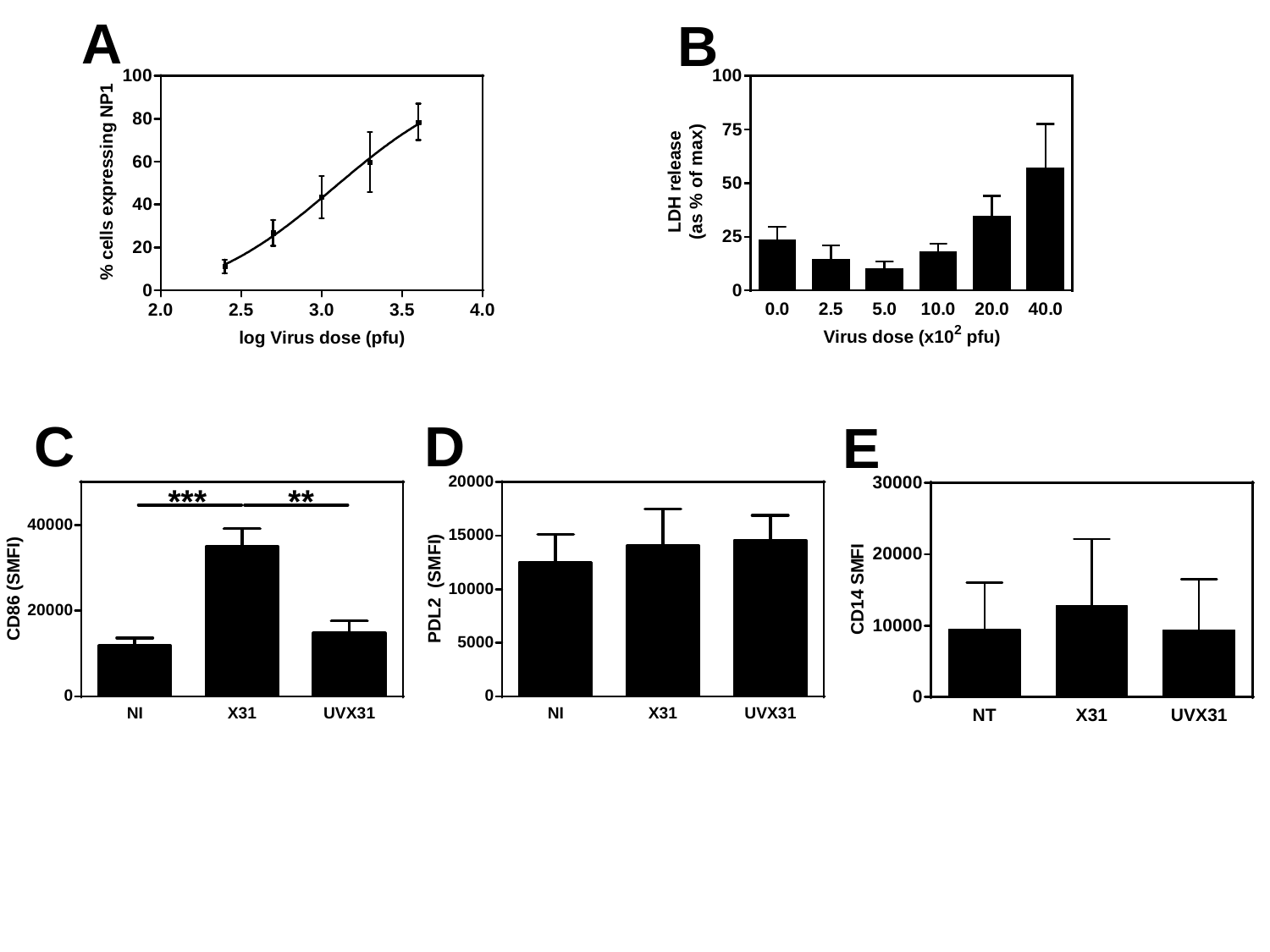

A
B
D
C
E

Supplement: S1 Fig — MDM were infected with increasing concentrations of X31 virus for 24 h and A) cells were analysed for viral NP-1 expression using flow cytometry and the percentage of NP1+ cells were gated using uninfected cells as a negative control. B) supernatants were harvested for LDH analysis. Data are expressed as means ± SE of 3 independent experiments. C—E) Histograms showing infected MDM expression of cell surface C) CD86 expression (specific mean fluorescence intensity—SMFI n = 4) and B) PDL2 expression (SMFI n = 7) E) CD14 expression (SMFI n = 4) expressed as means ± SE of n independent experiments. ** P<0.01, *** P<0.001 (PPTX) [file pone.0121527.s002.pptx]

## Slide 1
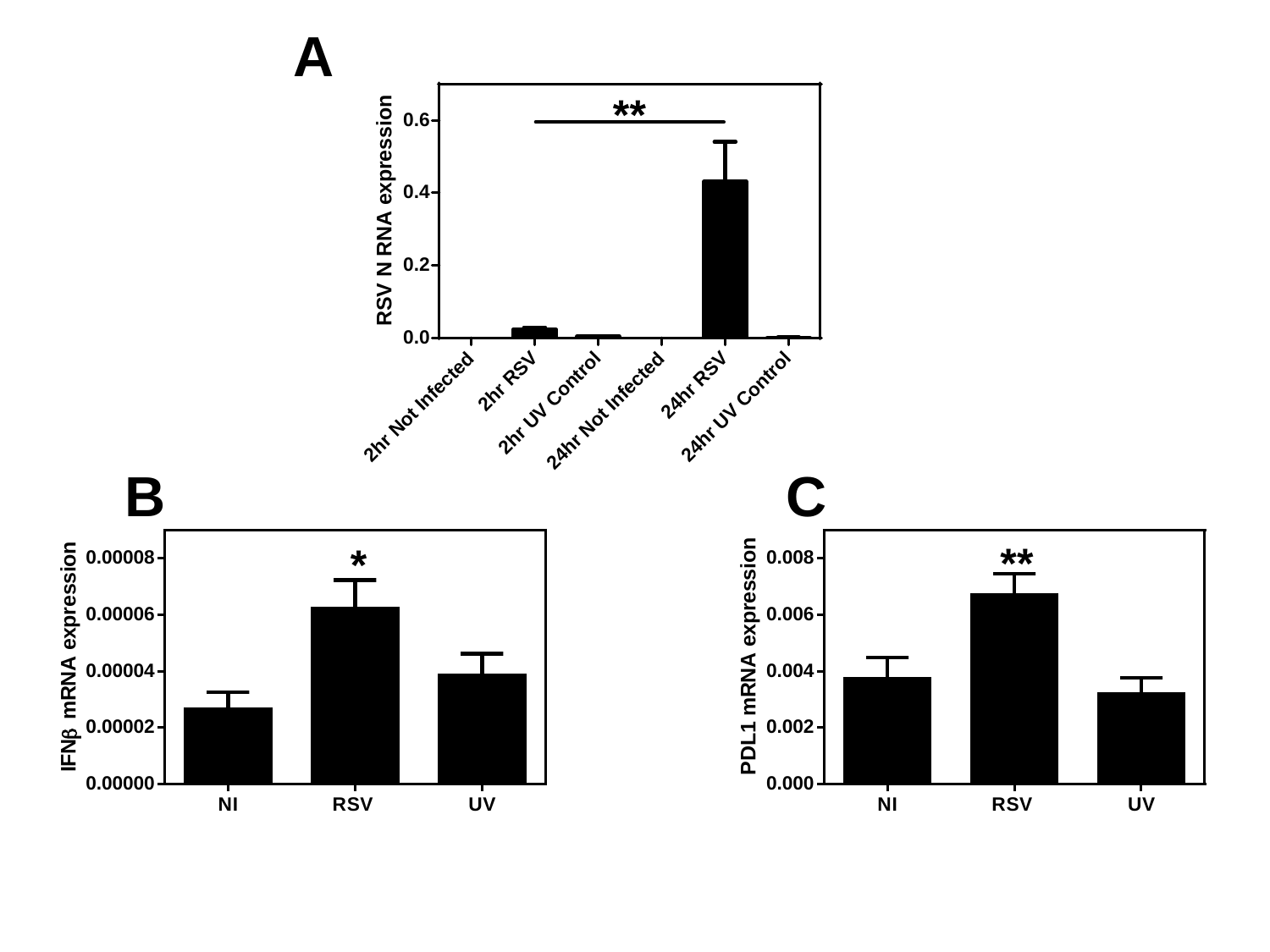

A
C
B

Supplement: S2 Fig — After infection with RSV, MDM were lysed and expression of A) RSV-N gene at 2 h and 24 h post infection, B) IFNβ gene expression at 24 h and C) PDL1 gene expression were measured by RT-PCR and normalized to b2-microglobulin. Data are expressed as mean ± SE 2^-delta Ct values of 7 independent experiments. * P<0.05, ** P<0.01. (PPTX) [file pone.0121527.s003.pptx]

## Slide 1
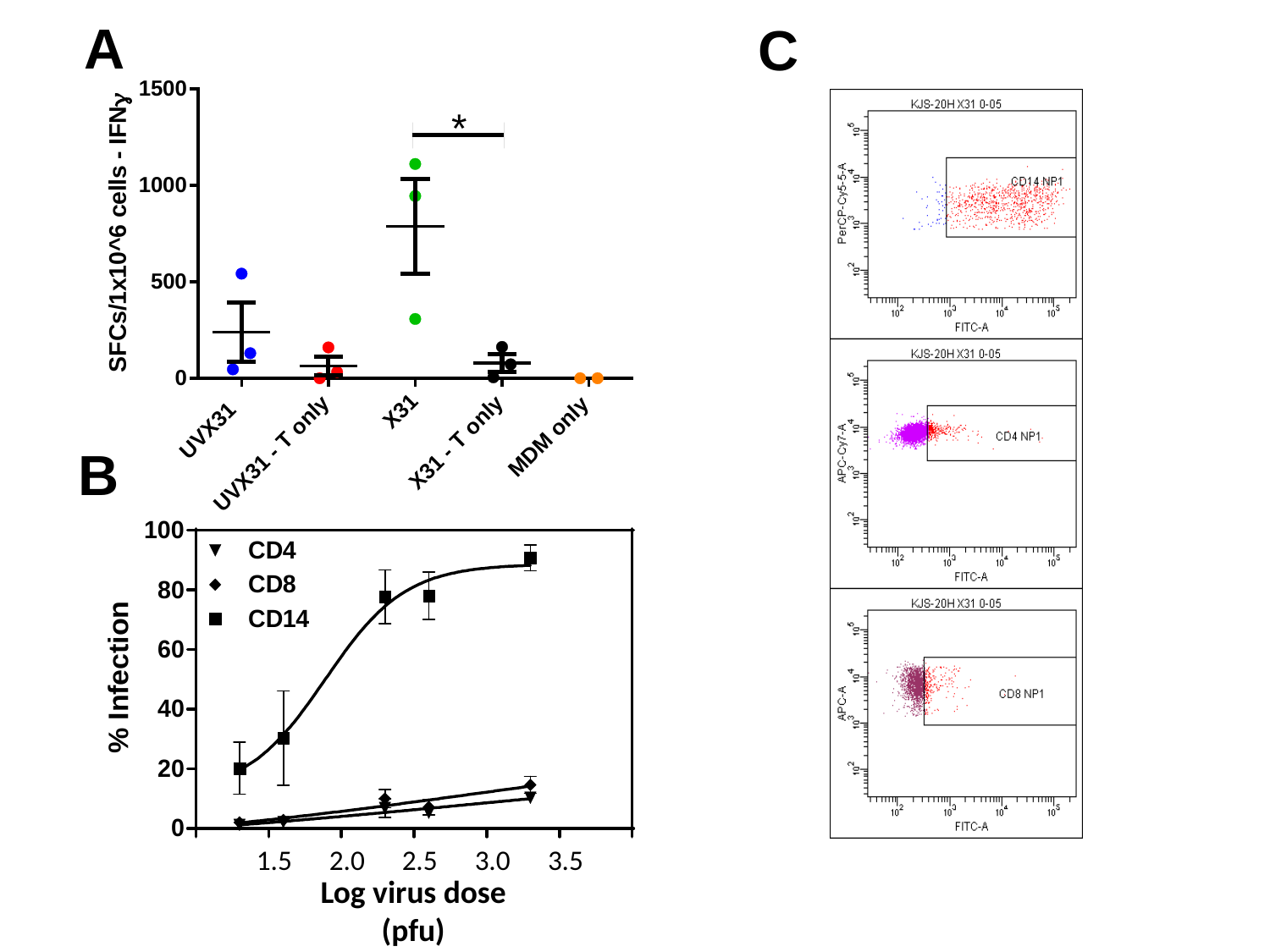

A
C
B
2.0
1.5
2.5
3.0
3.5
Log virus dose (pfu)

Supplement: S3 Fig — A) MDMs or lymphocytes were treated with UVX31 or X31 for 2 h before washing. 5 x 10^4^ MDMs were added to wells of an ELISPOT plate and were co-cultured with 2.5 x 10^5^ lymphocytes. Wells not containing MDMs (T only), and wells containing MDMs only were also measured for their ability to produce IFNγ. The ELISPOT plate was incubated at 37°C for 18 h before analysis. Mean and SEM shown of 3 independent experiments. Paired students t test was performed. p < 0.05 (*). B) Graph showing mean percentage (± SE n = 3 independent experiments) infection of CD14+ monocytes, CD4+ and CD8+ T lymphocytes fractions of PBMC cultures exposed to increasing concentrations of X31 influenza virus for 20 h as assessed by flow cytometry. C) Representative FACS plots of 3 independent experiments at the highest dose of virus used are shown. (PPTX) [file pone.0121527.s004.pptx]
